# Assessing the uncertainty around age-mixing patterns in HIV transmission inferred from phylogenetic trees

**DOI:** 10.1371/journal.pone.0249013

**Published:** 2021-03-25

**Authors:** David Niyukuri, Peter Nyasulu, Wim Delva

**Affiliations:** 1 Division of Epidemiology & Biostatistics, Faculty of Medicine and Health Sciences, Stellenbosch University, Cape Town, South Africa; 2 The South African Department of Science and Technology–National Research Foundation (DST-NRF) Centre of Excellence in Epidemiological Modelling and Analysis (SACEMA), Stellenbosch University, Cape Town, South Africa; 3 Division of Epidemiology & Biostatistics, School of Public Health, Faculty of Health, University of the Witwatersrand, Johannesburg, South Africa; 4 Center for Statistics, I-BioStat, Hasselt University, Diepenbeek, Belgium; 5 International Centre for Reproductive Health, Ghent University, Ghent, Belgium; 6 Rega Institute for Medical Research, KU Leuven, Leuven, Belgium; University of Ghana College of Health Sciences, GHANA

## Abstract

Understanding age-mixing patterns in Human Immunodeficiency Virus (HIV) transmission networks can enhance the design and implementation of HIV prevention strategies in sub-Saharan Africa. Due to ethical consideration, it is less likely possible to conduct a benchmark study to assess which sampling strategy, and sub-optimal sampling coverage which can yield best estimates for these patterns. We conducted a simulation study, using phylogenetic trees to infer estimates of age-mixing patterns in HIV transmission, through the computation of proportions of pairings between men and women, who were phylogenetically linked across different age groups (15–24 years, 25–39 years, and 40–49 years); and the means, and standard deviations of their age difference. We investigated also the uncertainty around these estimates as a function of the sampling coverage in four sampling strategies: when missing sequence data were missing completely at random (MCAR), and missing at random (MAR) with at most 30%—50%—70% of women in different age groups being in the sample. The results suggested that age-mixing patterns in HIV transmission can be unveiled from proportions of phylogenetic pairings between men and women across age groups; and the mean, and standard deviation of their age difference. A 55% sampling coverage was sufficient to provide the best values of estimates of age-mixing patterns in HIV transmission with MCAR scenario. But we should be cautious in interpreting proportions of men phylogenetically linked to women because they may be overestimated or underestimated, even at higher sampling coverage. The findings showed that, MCAR was the best sampling strategy. This means, it is advisable not to use sequence data collected in settings where we can find a systematic imbalance of age and gender to investigate age-mixing in HIV transmission. If not possible, ensure to take into consideration the imbalance in interpreting the results.

## Introduction

An age-disparate relationship is defined as a relationship where the male partner is 5 or more years older than the female partner [1]. At the population level, patterns of age-related sexual partner choices are known as age-mixing patterns [1]. In the same way, patterns of Human Immunodeficiency Virus (HIV) transmission across different age groups define the age-mixing patterns in HIV transmission.

The inter-generational transmission of HIV infection can make it persistent within populations. As explained by Beauclair [1], the *bridge width* (number of years difference between the maximum and the minimum partner age for someone in more than one sexual partnership) can explain an individual’s ability to transmit HIV infection to different age groups or generations. Explicitly, at a time point when an HIV positive individual is in two or more discordant relationships with large and small age-differences with his/her partners, this individual has the potential to transmit the infection between the two generations. The same can happen for individuals who have transient relationships with different age preferences over time.

In sub-Saharan Africa (SSA), the overall age and gender stratified prevalence of HIV shows a discrepancy between women and men [2, 3]. The fact that younger women of less than 25 years and those between 30–40 years have higher HIV prevalence rates than men of the same age categories [4] raises a great deal of concern. If these trends persist they will impede the efforts of having an HIV-free generation [4]. Age-disparate relationships can increase the risk of HIV transmission to younger women [5], and this may explain why the incidence of HIV infection among younger women in SSA is high [6].

To explain disproportionate prevalence observed in SSA among younger women, epidemiological and sexual behavior survey data have been used to study age-mixing patterns in sexual partnership and the risk of HIV transmission [7, 8]. However, the results of different studies analyzing the relationship between age-disparate relationships and the risk of acquiring an HIV infection in younger women have been contradictory.

On one hand, we have studies that concluded that there was no significant relationship between age disparity and the risk of HIV acquisition: Harling et al. [9], Balkus et al. [10], and Street et al. [11]. Harling et al. [9], they analyzed the sero-conversion data of a community-based cohort of women aged between 15 and 29 years collected from January 2003 to June 2012 in KwaZulu Natal. The age-disparity analysis of each woman’s most recent sexual partner at each round of HIV testing found that, it was not associated with subsequent HIV acquisition. Balkus et al. [10], they used data from 3789 South African women (18–45 years old) enrolled in the Vaginal and Oral Interventions to Control the Epidemic (VOICE) clinical trial between 2009 and 2012. The study found that reporting a partner >5 years older, or >10 years older was not associated with HIV acquisition. Street et al. [11], they used secondary data of a phase III multi-site, double-blinded, placebo-controlled trial, testing the safety and efficacy of the microbicide Carraguard^*TM*^, for the prevention of HIV infection in 1355 women aged 16 years and above between 2004–2007 (a 24 month follow-up study). The authors concluded that there was no significant relationship between age disparity and the risk of HIV acquisition.

On another hand, we have studies suggesting that age-disparate partnerships are a risk factor for HIV infection, including Evans et al. [5] and Akullian et al. [12], together with a phylogenetic study by De Oliveira et al. [13], where they looked at the population level proportion of transmission between age groups. With nationwide data sets from 2002, 2005, 2008, and 2012 for the South African National HIV Surveys, Evans et al. found that younger women with age-disparate partners had greater odds of being HIV positive in each survey year [5]. De Oliveira et al. [13], they performed a community-wide phylogenetic study in which the sequence data were collected in KwaZulu-Natal between June 2014 to June 2015. The results suggested that a phylogenetic linkage between younger women and older men depicted a transmission cycle, i.e., younger women obtained the infection from older men and once they became adults, they transmitted the infection to men of the same age group, and these men, in turn, transmitted the infection to other younger women. Thus, they continued the cycle. Akullian et al. [12], they used a cohort data set from KwaZulu Natal (2004–2015), estimating the smoothed HIV incidence rates across partnership age pairings between men and women, and the relative risk of HIV acquisition by the partner. The study found that the age of the sexual partner was a major risk factor for HIV transmission in both men and women. This confirmed the HIV transmission cycle driven by age difference between men and young women in the study of De Oliveira et al. [13].

A particular attention should be paid to the use of viral sequence data to bring a clear understanding of age-mixing patterns as a major factor that increases the spread of HIV in SSA [13]. It is an objective and promising approach as it reduces the bias associated with recall, and sexual behavior surveys, mainly the social desirability biases [14–17].

The objective of this simulation study was to investigate whether age-mixing patterns in HIV transmission can be inferred from phylogenetic trees through the computation of the proportions of men/women of different age groups phylogenetically linked to women/men known as pairings; and the means, and standard deviations of their age difference. In addition, in the same way that sample size, and sampling strategy have effects on the estimates from surveys, we explored how scenarios of missingness of sequence data (referred to sampling strategies) and the sampling coverage (referred to sample size) affect the proportions of pairings, and the mean, and standard deviation of age difference between men and women who are phylogenetically linked. That exploration, suggested the best sampling strategy (data missingness scenario), and sub-optimal threshold of sampling coverage.

## Materials and methods

In this simulation study, we considered a population of men and women within a generalized HIV epidemic in a heterosexual network. The simulation was conducted using agent-based models (ABMs) with Simpact Cyan 1.0 simulation tool [18], which simulated dynamic sexual network, HIV transmission dynamic, and viral evolution across the transmission network. More explicitly, the dynamic of sexual network is simulated through establishment and dissolution of sexual partnerships. Sexual partnership and dissolution events occurrence rates are given by their hazard functions, which are mathematical quantities which depend mainly on age of individuals, age of their partners, ongoing relationship if there is any, among others factors. Within the partnership network, HIV transmissions occur as a function of several factors, i.e., the partner’s HIV status, viral load levels, antiretroviral treatment (ART) intervention, and follow-up of the HIV positive partner. In addition to these mentioned events, other demographic events, including birth and death, were considered, and behaviours of infected individuals (diagnosis and ART intervention) were also recorded. The simulation platform provided a full control of the data generation process and, hence, provided a platform to measure age-mixing patterns in HIV transmission network, and the uncertainty around estimates of those patterns inferred from phylogenetic trees. More details on set of the Simpact Cyan 1.0 simulation tool, the simulation work-flow, parameters from events’ hazard functions and related settings can be seen in the S1 Appendix. The first and second tables in the S1 Appendix are a recapitulation of parameters, and key assumptions which were considered to produce the sexual network, and HIV transmission network data. The third table in the S1 Appendix describes the evolutionary dynamic of HIV in our simulation.

### HIV epidemic simulation

Simpact Cyan simulation framework has a lot of parameters which are set at default based on common knowledge and evidence from the literature for sexual partnership, HIV transmission, and viral evolution. By tweaking some of these parameters we can be able to mimic different epidemic trends, such as those observed in generalized HIV epidemic settings [19–21] and some sexual behavior related to sexual partnership in Southern Africa [7, 8, 22, 23]. In our case, an HIV epidemic was simulated in an age- and gender-structured population. With an initial population of 10,000 men and 10,000 women, the simulation time was 40 years, and HIV infection was introduced in the population at the 10th year among 10 randomly selected individuals, whose age ranged between 20 and 50 years. During the simulation, different events which controlled the interactions of agents occurred at different rates as described in the S1 Appendix. Treatment eligibility based on Cluster of Differentiation 4 (CD4) counts was gradually factored in the simulation as described in the S1 Appendix.

For molecular evolution, to simulate viral sequence data for infected individuals, we used a full transmission tree of infected individuals and a root sequence data. Each seed individual who introduced HIV has his/her own transmission network which was transformed in a transmission tree using *epi*2*tree* function of the R package *expoTree* [24]. We combined recursively all the transmission trees of the seed individuals (10), and built one transmission tree. We transformed the new tree into a binary tree using the *multi*2*di* function of the R package *phytools* [25]. The final tree was used for a forward simulation of substitutions of the viral sequences (each per individual) using the *GTR* + Γ substitution model in Seq-Gen [26]. The root sequence was an HIV-1 sub-type C [27], and for simplicity we considered only the polymerase (POL) gene [28].

In order to build time-stamped phylogenetic tree, we projected the simulation time to calendar time by assuming that the simulation of sexual partnership started in 1977, and HIV introduction was done in 1987, 10 years after, and the end of simulation was 2017, which was 40 years of simulation time.

At the time point of 40 years of simulation time (2017 of calendar time), the epidemic was characterized by an increasing prevalence across low age groups in both men and women, with women carrying a disproportionate burden (S1 Fig). And between 35–40 years of simulation time, younger women (below 25 years) had higher incidence compared to men of the same age group (S2 Fig).

### Estimating HIV transmission network and proportions of HIV transmission pairings

From simulated sequence data, after computing a time-stamped phylogenetic tree of a sampled population using FastTree [29] software and the R package *treedater* [30], we identified transmission clusters based on high support for the grouping and low within-cluster genetic distance using the Cluster Picker software [31].

Estimating the transmission network from the phylogenetic trees was based on estimating HIV transmission pairings within transmission clusters, by using the time to the most recent ancestor matrix (tMRCM) [32], and the characteristics of individuals in transmission clusters, mainly gender and age [13]. We first computed the time to the most recent ancestor matrix (tMRCA), which was a contingency matrix. Thereafter, we filtered this matrix by gender, transmission cluster identifier, and a threshold value of tMRCA at 7 years [32]. Thus, we obtained a pairing between individuals *x*_*i*_ and *x*_*j*_ if they were within same transmission cluster, had different gender, and the tMRCA between them did not exceed 7 years. Note that an individual can be connected to more than one individual.

Similarly to De Oliveira et al. [13], the age groups we considered in this simulation study were less than 25 years, 25–39 years, and 40–49 years for men and women. In our analysis, we considered the proportions of women who were phylogenetically linked to men: (i) women between 15 and 24 years and men of the same age group, (ii) women between 15 and 24 years and men between 25 and 39 years, (iii) women between 15 and 24 years and men between 40 and 49 years, (iv) women between 25 and 39 years and men of same age group, and (v) women between 25 and 39 years and men between 40 and 49 years. From men perspective, we computed the proportions of men who were phylogenetically linked to women: (i) men between 15 and 24 years and women of the same age group, (ii) men between 25 and 39 years and women between 15 and 24 years, (iii) men between 25 and 39 years and women of the same age group, (iv) men between 25 and 39 years and women between 15 and 24 years, and (v) men between 40 and 49 years and women between 25 and 39 years.

Besides, proportions of pairings, we computed also the means and standard deviations of the age difference [33], between men and women in transmission clusters. To compute the age difference, we considered the age difference between men/women in any age group (15–24, 25–39, and 40–49 years) and their pairs women/men phylogenetically linked together regardless of the age group. This provided information on the magnitude of age gap in HIV transmission across different age groups.

### Age mixing patterns in sexual partnerships

To be able to evaluate our results, we computed true age-mixing patterns in sexual partnerships which made the sexual network across which HIV infection was transmitted. We simulated age disparity relationship by setting age-gap preference parameters’ values for sexual partnership. We assumed that age gap was drawn from a normal distribution with 10 years and 5 years for the mean and standard deviation of the age gap, respectively, as shown in the first table of parameters’ values in the S1 Appendix.

If a male individual with age *i* is (or has been) in sexual partnership with *n* women, with each of them having age $a_{i}^{j}$ (with *j* ∈ [1, *n*]), the age-mixing patterns within the general sexual network can be explained by descriptive statistics of age difference, namely the average age difference (AAD) across relationships, and the standard deviation of these age difference (SDAD). The AAD is the mean of age gap across men’s sexual partnerships, and the SDAD is the standard deviation of age gap across men’s sexual partnerships. More than that, given the nature of the sexual partnerships data (clustering data), we can use a Linear Mixed-Effects Model (LMM) [34] to investigate the age-mixing patterns in sexual partnerships.

For any man *i* with *n* partnerships, there are *n* values of age gap preferences, thus, we had a clustered data set where the clustering unit was the man. If we consider a linear mixed random-effect model [34], to explain the variation of man’s age gap preference for his women partners, for a man *i*, the fitted LMM model was

$$\begin{array}{c} y_{ij}=\beta_{0}x_{ij}+\beta_{1}+b_{i}+\epsilon_{ij} \end{array}$$

where *y*_*ij*_ represents the age gap preference of woman *j* in sexual partnership with man *i*, and *x*_*ij*_ was the predictor which was the age of the man *i*. The parameters *β*_0_ and *β*_1_ represented the fixed effects, while *b*_*i*_ parameters represented the random effects. We fitted the Linear Mixed-Effects Models Using the R package *lme*4 [35].

Thus, from the model outputs, we have the within-subject standard deviation of age differences (WSD), the average variation of age gap within the clusters of men’s age gaps; the between-subject standard deviation (BSD), the average variation of age gap between the clusters of men’s age gaps. The overall population level trend of age difference was also depicted by the slope and intercept of the LMM model.

### Data missingness scenarios

By assuming that the uncertainty around age-mixing (inferred from transmission clusters) may be associated with sequence missingness, and low sampling coverage, we explored different missingness scenarios and sampling coverage, to determine the best missingness scenario, and a sub-optimal sampling coverage.

The missingness of sequence data is not like the missingness of a data point in a data table [36], but some mechanisms of data points missingness can be applied to missingness of sequence data. Thus, we considered two main mechanisms of data missingness scenarios for viral sequence data: missing completely at random (MCAR) and missing at random (MAR) [37]. Each missingness scenario explained a sampling strategy which can happen when collecting sequence data. If sequence data are missing completely at random (MCAR), this means that the missing observations (sequences) are a random subset of all observations. Missing completely at random (MCAR) indicates that there was not a systematic procedure to make certain sequence data more likely to be missing than others [37]. For sequence data missing at random (MAR), there might be a systematic difference between these missing sequences and the ones we observed in our sample. If sequence data are missing at random (MAR), conditional on age and gender, then the distributions of missing and observed sequence data will be similar among people of the same age and gender [37]. Thus, for missing at random (MAR), we assumed there was differences of sample proportions among different age groups and gender. We considered MAR scenarios where we had at most 30%, 50%, and 70% of women in the sample in each of the three age groups (less than 25 years, 25–39 years, and 40–49 years). Therefore, in total, we had 4 scenarios of data missingness: one for MCAR, and three for MAR.

In each of the 4 sampling strategies (data missingness scenarios), we had 13 sampling coverages (from 35% to 95% with an interval of 5%).

We compared the difference between estimates from the two types of sampling strategies (MCAR and MAR) at different sampling coverages using the Wilcoxon test [38], since simulation outputs were not normally distributed. The null hypothesis is that the vectors of the parameter values in MCAR and MAR were from the same distribution. This was rejected when the p-value was less than the 0.05 significance level. For the 2800 simulations, each parameter had 4 vectors of values (one for MCAR, and three for MAR scenarios: with 30%, 50%, and 70%) at each sampling coverage. The use of the Wilcoxon test tells us whether the median values of the two-by-two comparison of the parameter values were from same continuous distribution or not. The comparison was made between the MCAR scenario and MAR scenarios (having at most 30%, 50%, and 70% women in the sample).

The workflow of the study design was elaborated as follows: (i) we simulated an HIV epidemic within a heterosexual network, (ii) we simulated the evolutionary dynamics of the virus across transmission networks, (iii) we defined a sampling strategy and constructed a phylogenetic tree of sequences from sampled individuals within a time interval of 35–40 years simulation time, (iv) we computed the transmission clusters from the phylogenetic tree, (v) then, we estimated a transmission network with pairings by filtering the entire time to the most recent common ancestor (tMRCA) matrix from the phylogenetic tree by gender, transmission cluster identifier, and time to most recent common ancestor, (vi) we computed the proportions of men/women in different age groups in partnership with women/men of certain age groups within the transmission clusters, (vii) we computed the age difference statistics (mean and standard deviation) of people within transmission clusters, (viii) we analyzed the best sampling strategy and sub-optimal sampling coverage by computing the root mean square error (RMSE), between true values of the proportions of pairings and age difference statistics between (obtained at 100% sampling coverage), and those from inference.

To count for stochasticity, with the same parameter combination, we ran 2800 simulations. To summarise the estimates of proportions of the pairings; mean, and standard deviation of the age difference; and statistics of age-mixing in sexual partnership obtained in any of the 4 scenarios at any sampling coverage among the 13 (per scenario), we considered their median values, since for each estimate at every sample coverage we had 2800 data points.

All estimates were computed from data sampled between 35–40 of simulation time in different sampling strategies, and sampling coverages. All scripts to reproduce the results, and data generated, are publicly available at a GitHub repository (https://github.com/niyukuri/age_mixing_patterns_phylogenetic).

## Results

### Proportions of phylogenetically linked pairings across age groups

Between 35 and 40 year of simulation time, the median values of number of true pairings (number of pairs of men/women phylogenetically linked together to women/men) in the transmission network for all HIV positive individuals were given in the Table 1. By descending order of phylogenetic pairings in age groups, we have: men aged 25–39 years with women aged 15–24 years (30 pairs), followed by men aged 40–49 years and women aged 15–24 years (15 pairs), men aged 40–49 years and women aged 25–39 years (14 pairs), men aged in 25–39 years and women of the same age group (six pairs), and men aged 15–24 years and women of the same age group (six pairs). We had zero median value for pairs between men aged 15–24 years and women aged 25–39 years, men aged 15–24 years and women aged 40–49 years, men aged 25–39 years and women aged 40–49 years, and men aged 40–49 years and women aged 40–49 years.

**Table 1:** Phylogenetic pairings at 100% sampling coverage within 35–40 years of simulation time.

		**Women**		
		**15–24 years**	**25–39 years**	**40–49 years**
**Men**	**15–24 years**	6	0	0
	**25–39 years**	30	6	0
	**40–49 years**	15	14	0

**Fig 1:**
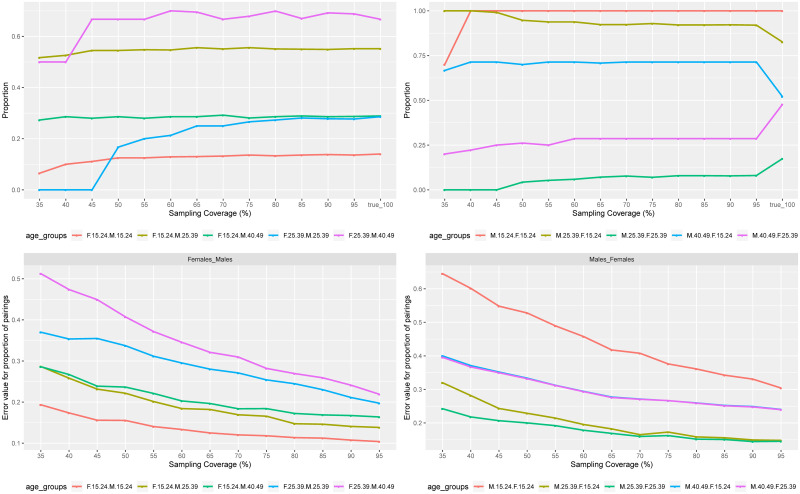
Median values of proportions of pairings in different age groups and precision error as a function of sample coverage. On the top left hand side is the proportion of women in age group A phylogenetically linked to men in age group B, and on the right hand side is the proportion of men in age group B linked to women in age group A. At the bottom left and right hand side, are the differences between the true values of the proportions of pairings at 100% coverage and those obtained from different missing completely at random (MCAR) sampling coverages for different age group linkages between women and men.

At the top left hand side of Fig 1, for the MCAR sampling strategy, on average, the proportion of women aged 15–24 years who were phylogenetically linked to men of the same age group was low (around 10%) compared to the proportion of men aged 15–24 years who were phylogenetically linked to women of the same age group (100%) at the the top right hand side. At the same figure, across all sampling coverages, around 55% of younger women (15–24 years) were phylogenetically linked to men between 25 and 39 years, and 28% of these younger women (15–24 years) were phylogenetically linked to men between 40 and 49 years old. But, men aged 25–39 years and 40–49 years were phylogenetically linked to younger women (15–24 years) at around 90% (the true value was around 80%) and 70% (the true value was around 50%) proportions, respectively. Although these values were 10% and 20% greater than the true values, they had a steady trend from the 50% sampling coverage and above.

For women in the 25–39 years of age group, on average, around 67% of them were phylogenetically linked to men between 40 and 49 years old, and, on average, around 25% of these women were phylogenetically linked to men of the same age group (25–39 years). The proportions of men aged 40–49 years, and 25–39 years who were phylogenetically linked to women aged 25–39 years were on average around 27% and 7%, respectively, across all sampling coverages, but the true values were 47% and 17%, respectively. The trend of the proportions of pairings between women of the 25–39 years of age group and men aged 25–39 years and 40–49 years was quasi-symmetric.

By comparing the two figures of proportions of pairings between women and men across age groups at the top of Fig 1, as the sampling coverage increased, the estimates were improved towards the true values for the proportions of women of any age group phylogenetically linked to men in any other age group. On average, starting at 55% of the sampling coverage, the proportions of women phylogenetically linked to men were already close to the true values observed at 100%. However, this was not the case for the proportions of men of any age group who were phylogenetically linked to women, even at 95% of sampling coverage, the estimates were far from the true values as observed at the right hand side on the top of Fig 1.

In terms of the magnitude of the proportion values for women phylogenetically linked to men, on average, the first was for women between 25 and 39 years linked to men aged 40–49 years, followed by women aged 15–24 years linked to men aged 25–39 years, women aged 15–24 years linked to men aged 40–49 years, women aged 25–39 years linked to men of the same age group, and women aged 15–24 years linked to men of the same age group.

For the proportions of men phylogenetically linked to women, the highest magnitude was for men aged 15–24 years linked to women of the same age group, followed by men aged 25–39 years linked to women aged 15–24 years, men aged 25–39 years linked to women of the same age group, men aged 40–49 years linked to women aged 25–39 years, and men aged 40–49 years linked to women aged 15–24 years.

For the RMSE values, between the true proportions at 100% of the sampling coverage and those in different sampling coverages of the MCAR sampling strategy, we observed a decreasing trend as we increased the sampling coverage as seen at the bottom of Fig 1. The best performance was for the proportions of women linked to men (left hand side at the bottom of Fig 1), if we compared to men linked to women (right hand side at the bottom of Fig 1).

For the sampling strategy, where missing sequences were missing at random (MAR), the trend of the proportion values across the sampling coverages in all three MAR scenarios (with at most 30%, 50%, and 70% of women were in the samples) were different from the MCAR scenario, which may be explained by the age group and gender imbalance in the samples. The overall trends of the RMSE for the proportions decreased when the sampling coverage increased in all three scenarios, where we assumed that at most 30%, 50%, and 70% of women were in the sample.

**Fig 2:**
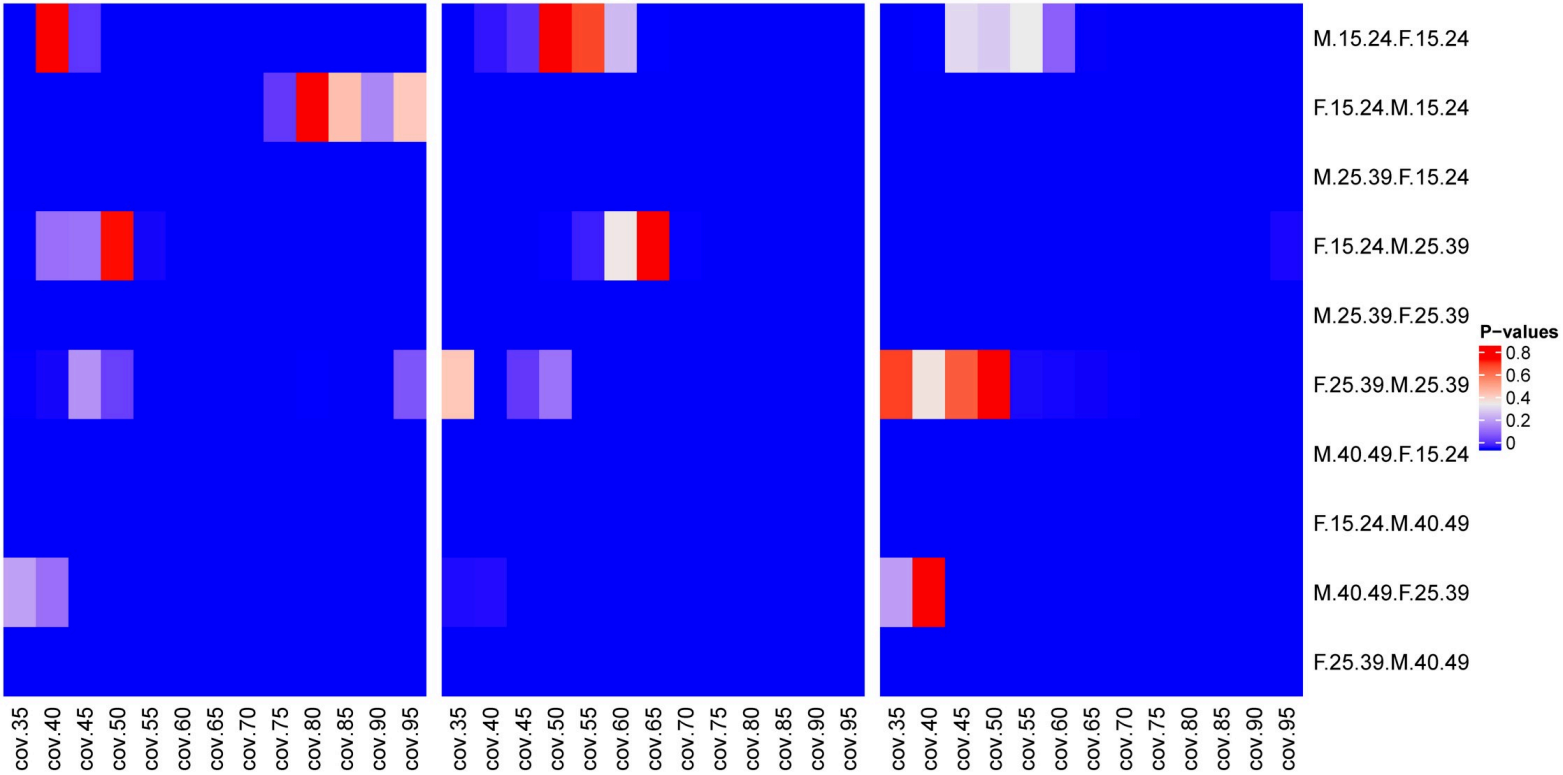
Comparison of median values for proportions of pairings in different age groups for three sampling strategies as a function of sampling coverage. The P-values of the Wilcoxon signed-rank paired test between the proportions of pairings across age groups at different sampling coverages between MCAR and missing at random (MAR) missingness scenarios: MCAR and MAR with at most 30% of women in the sample (left hand side), MCAR and MAR with at most 50% of women in the sample (in the middle), and MCAR and MAR with at most 70% of women in the sample (right hand side).

The comparison between the median values of proportions of pairings in MCAR scenario and in MAR three scenarios by the Wilcoxon test at Fig 2, showed that the majority of the median values of the proportions of pairings across different age groups between men and women were from different distributions.

The Fig 2 shows that, at higher sampling coverages (above 65%), except for the proportions of younger women (15–24 years) phylogenetically linked to men of the same age group in MCAR and in MAR with at most 30% of the sample being women, the median values of the other parameters were from distinct distributions in all comparisons. For comparisons with sampling coverages below 65%, we found sporadic cases, where we could conclude that the proportions of pairings came from same distributions. However, the predominant scenarios were when we had proportions from different distributions. The cases where we have median values of proportions of pairings from same distributions may be explained by sparse sampling.

### Age difference in phylogenetically linked pairings from transmission clusters

**Fig 3:**
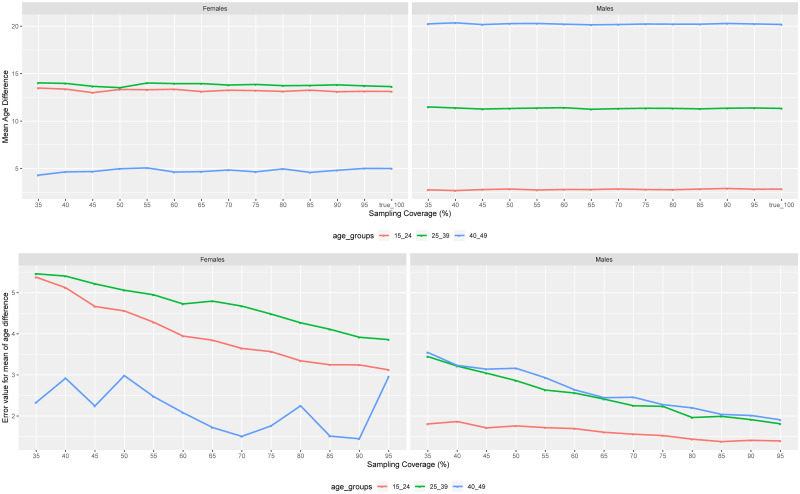
Median values of means of age difference between pairings in different age groups as a function of sampling coverage. The values of the mean age difference within pairings across age groups as a function of the sampling coverage (on the top) for MCAR sampling strategy. And the difference between true values of the mean age difference at 100% coverage and those obtained from different MCAR sampling coverages (at the bottom) as a function of sampling coverage.

In the MCAR sampling strategy, the trend of the mean age difference for women and men in almost all age groups appeared to be steady across all sampling coverages. These estimates did not greatly deviate from the true values as we can see at the top of Fig 3.

The highest magnitude of women’s age difference was observed for women aged 25–39 years, followed by women aged between 15 and 24 years, with their age gap being between 13 and 14 years. The lowest age difference was for women aged 40–49 years old (around 5 years). Compared to men, the highest mean age difference was for men aged 40–49 years old (around 20 years), followed by men aged 25–39 years old (around 11 years), and men aged 15–24 years old (around 2 years).

Comparing the results to the true values of the mean age difference at 100% coverage, the RMSE values showed a decreasing trend as we can see at the bottom of Fig 3. The decrease of the error appeared to be faster for men compared to women in all age groups. The error associated to the age difference for women aged 40–49 years had sporadic behavior across the sampling coverage but did decrease. The error values for the means of the age difference were reported to be between 0 and 5 years, and 0 and 3 years for women and men, respectively (see the bottom of Fig 3).

**Fig 4:**
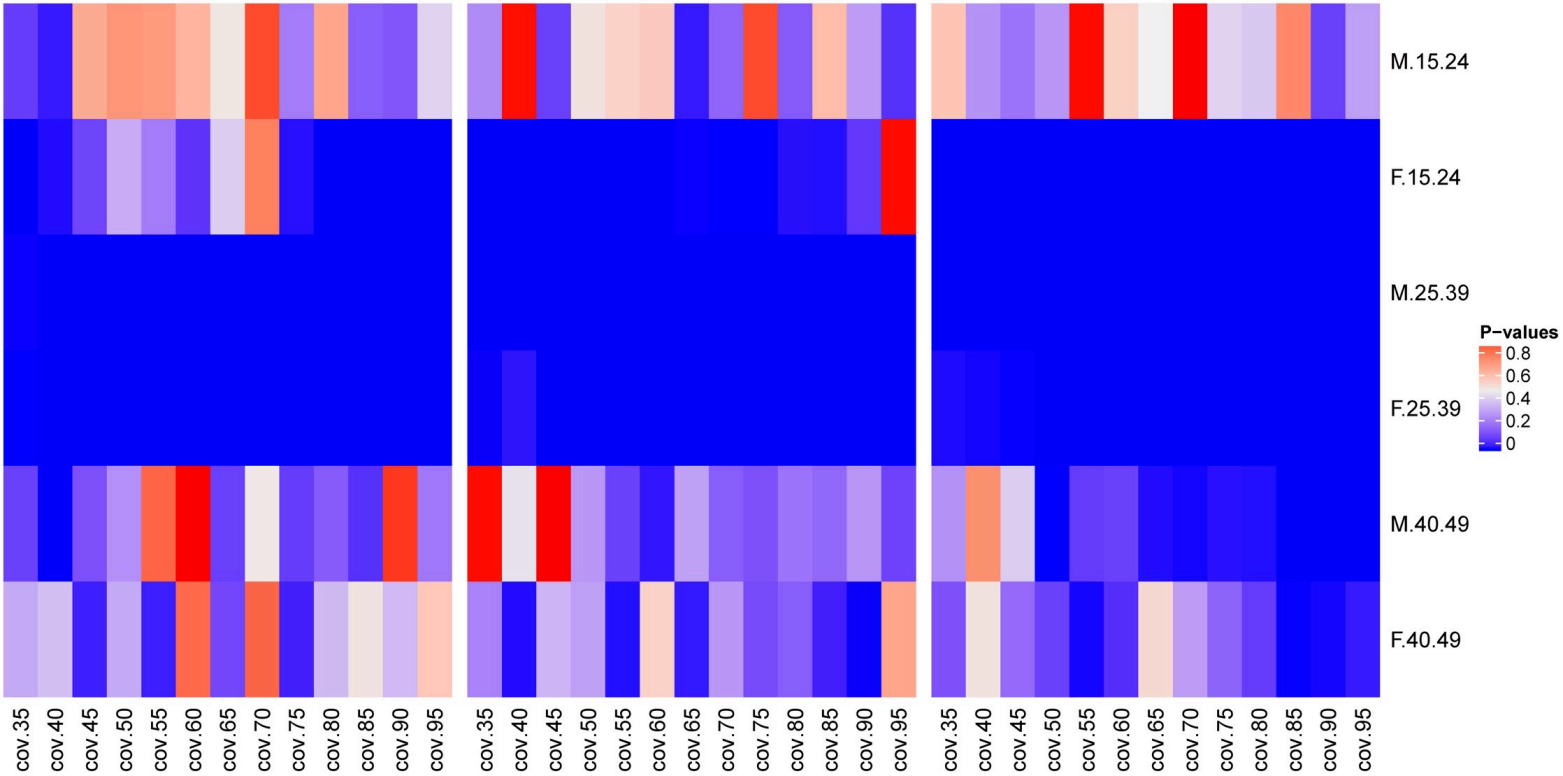
Comparison of median values for means of age differences between pairings in different age groups as a function of sampling coverage. The P-values of the Wilcoxon signed-rank paired test between the means of age difference in pairings at different sampling coverages between the MCAR and MAR missingness scenarios: MCAR and MAR with at most 30% of women in the sample (left hand side), MCAR and MAR with at most 50% of women in the sample (in the middle), and MCAR and MAR with at most 70% of women in the sample (right hand side).

If we compare the means of the age difference across the age groups in the MCAR and MAR scenarios (see Fig 4), We can see that the means age differences for men with 25–39 years of age and women of the same age group, and women below 25 years did not come from the same distributions. However, for men aged 15–24 years and those aged 40–49 years of age, and women with 40–49 years of age, the majority of their sampling coverage comparison demonstrated that their means age difference values came from same distributions. But in those age groups we had even small sampling coverage sporadic cases where the means age difference were from different distributions.

**Fig 5:**
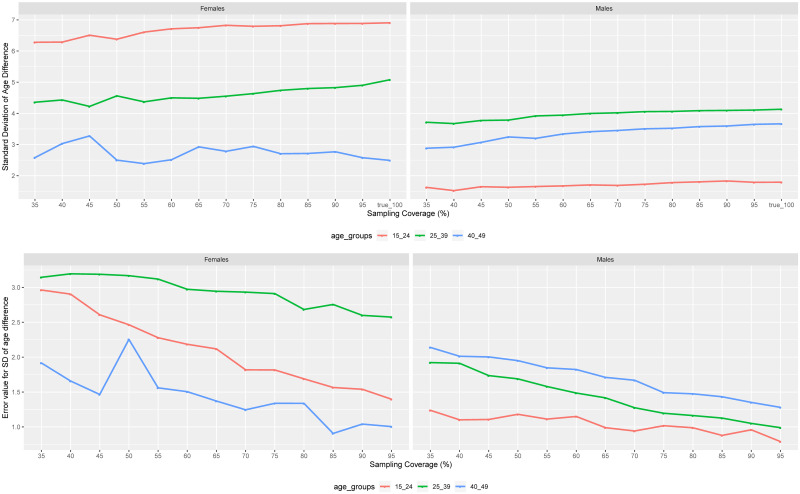
Median values of standard deviations of age difference between pairings in different age groups as a function of sampling coverage. The values of the standard deviation of the age difference within pairings across age groups as a function of the sampling coverage (on the top) for MCAR sampling strategy, and the difference between true values of the standard deviation of age difference at 100% coverage and those obtained from different MCAR sampling coverages (at the bottom) as a function of sampling coverage.

In the same sampling strategy for MCAR, the standard deviation of the age difference for women and men in almost all age groups improved as we increased the sampling coverage (top of Fig 5). Compared to women, men had the lowest standard deviation values in all age groups. The standard deviation values showed the dispersion of the age difference. For women, the highest magnitude of the standard deviation was for younger women (almost 7 years), followed by women aged 25–39 years (around 5 years), and women aged 40–49 years (around 2.5 years). For men, the highest magnitude of standard deviation was for men aged 25-39 years (around 4 years), followed by men aged 40–49 years (around 3.5 years), and younger men (around 1 year).

Compared to the true values of age difference standard deviations at 100% coverage, the RMSE decreased as we increased the sampling coverage (bottom of Fig 5). However, as previously seen for the means of age difference, the error values of the age difference standard deviation for women decreased slowly compared to men. The error values for the standard deviation of the age difference were reported to be between 0 and 3 years, and 0 and 2 years for women and men, respectively (the bottom of Fig 5).

**Fig 6:**
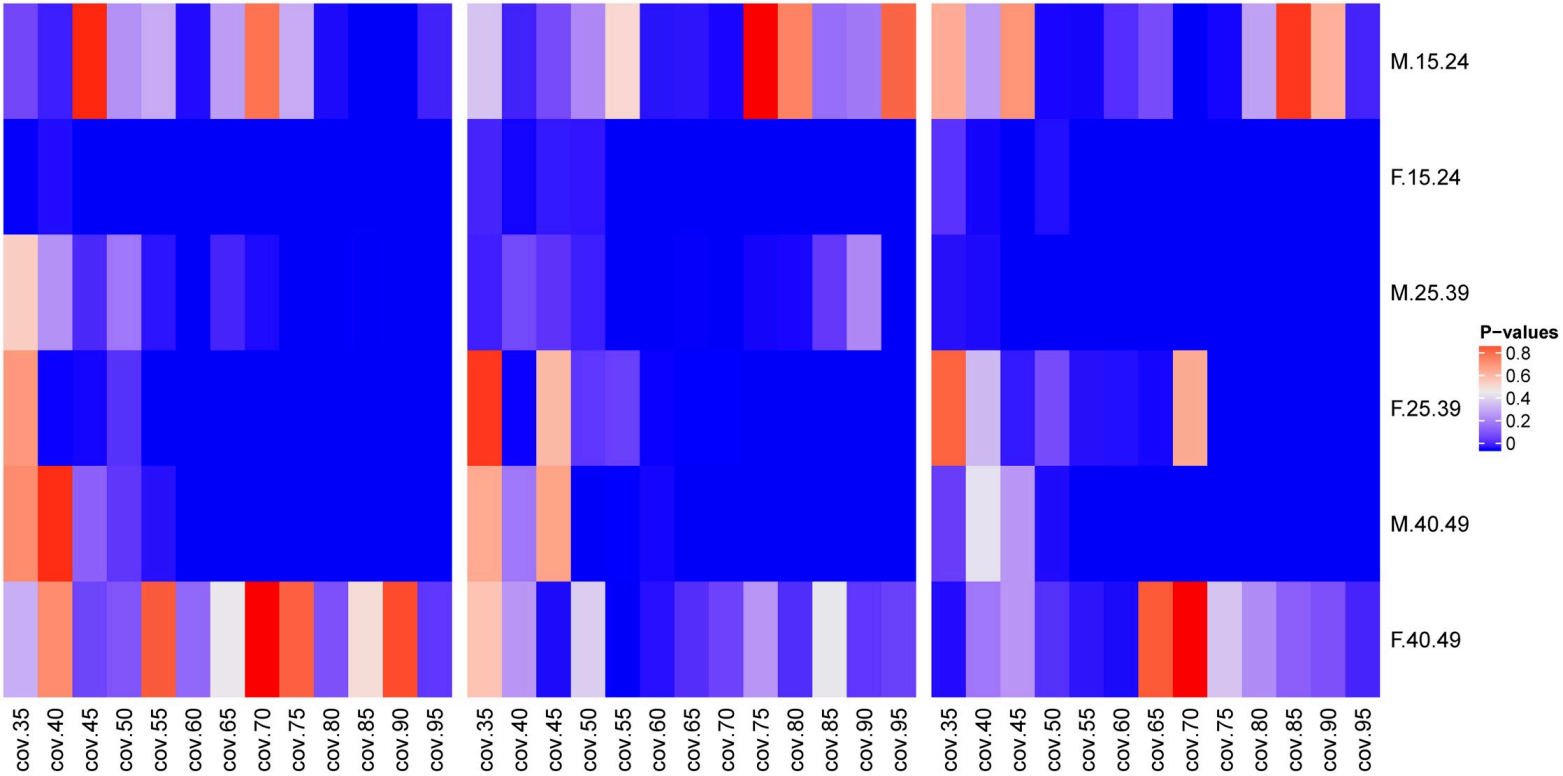
Comparison of median values for standard deviations of age differences between pairings in different age groups as a function of sampling coverage. The P-values of the Wilcoxon signed-rank paired test between the standard deviations of the age difference in pairings at different sampling coverages between the MCAR and MAR missingness scenarios: MCAR and MAR with at most 30% of women in the sample (left hand side), MCAR and MAR with at most 50% of women in the sample (in the middle), and MCAR and MAR with at most 70% of women in the sample (right hand side).

If we compare the standard deviations of the age differences across age groups in the MCAR and MAR scenarios (Fig 6) showed the same trend as the mean age difference. For men with 25–39 years of age and women of the same age group, their standard deviations did not come from the same distribution. However, certain other age groups: men of 15–24 years of age, men of 40–49 years of age, and women of 40–49 years of age, the majority of the sampling coverage showed that their standard deviation values came from the same distribution.

### Age-mixing patterns in sexual partnerships of infected individuals

In all 4 data missingness scenarios, the true median values of parameters of age-mixing in sexual partnerships (of infected individuals) were computed from the recorded data for 5 years (35–40 simulation years). The Table 2 shows the median value for the average age difference (AAD) across sexual partnerships, the standard deviation of the age difference (SDAD), the between-subject standard deviation of age differences (BSD), the within-subject standard deviation of age differences (WSD), and the slope and the intercept from the linear mixed effect model for age difference preference for men.

**Table 2:** Parameters of age mixing in sexual partnerships of infected individuals at different sampling coverage (%) when missing individuals were missing completely at random (MCAR), missing at random (MAR) with at most 30%, 50%, and 70% women in the sample.

Scenario	Parameter	35%	40%	45%	50%	55%	60%	65%	70%	75%	80%	85%	90%	95%	True at 100%
MCAR	AAD	13.188	13.16	13.161	13.13	13.146	13.135	13.117	13.128	13.137	13.145	13.128	13.129	13.121	13.918
	SDAD	6.346	6.386	6.372	6.401	6.378	6.387	6.402	6.419	6.431	6.429	6.427	6.431	6.429	6.201
	BSD	1.752	1.751	1.738	1.758	1.756	1.75	1.745	1.744	1.752	1.744	1.746	1.752	1.752	2.298
	WSD	1.684	1.691	1.696	1.706	1.694	1.7	1.702	1.699	1.701	1.699	1.706	1.702	1.705	1.787
	Slope	0.26	0.259	0.258	0.26	0.26	0.26	0.259	0.257	0.259	0.26	0.258	0.26	0.259	0.333
	Intercept	-1.949	-1.921	-1.946	-1.95	-1.956	-1.948	-1.951	-1.95	-1.938	-1.941	-1.952	-1.956	-1.93	-2.581
MAR (30% women)	AAD	11.203	11.449	11.673	11.85	11.992	12.156	12.236	12.347	12.461	12.514	12.61	12.703	12.779	13.918
	SDAD	6.734	6.659	6.596	6.559	6.507	6.476	6.436	6.404	6.392	6.386	6.385	6.4	6.401	6.201
	BSD	1.487	1.519	1.533	1.548	1.557	1.583	1.587	1.6	1.613	1.633	1.643	1.664	1.685	2.298
	WSD	1.667	1.67	1.667	1.671	1.671	1.67	1.671	1.675	1.676	1.679	1.681	1.688	1.693	1.787
	Slope S	0.201	0.208	0.212	0.219	0.222	0.227	0.23	0.233	0.235	0.238	0.241	0.245	0.247	0.333
	Intercept	-0.871	-1.006	-1.108	-1.207	-1.283	-1.352	-1.406	-1.483	-1.517	-1.569	-1.616	-1.686	-1.742	-2.581
MAR (50% women)	AAD	10.689	10.883	11.079	11.258	11.43	11.596	11.724	11.819	11.965	12.048	12.167	12.235	12.329	13.918
	SDAD	6.82	6.797	6.777	6.718	6.666	6.623	6.569	6.555	6.525	6.496	6.456	6.43	6.407	6.201
	BSD	1.425	1.436	1.469	1.489	1.523	1.537	1.551	1.544	1.565	1.576	1.589	1.59	1.601	2.298
	WSD	1.661	1.659	1.665	1.669	1.668	1.674	1.672	1.67	1.676	1.669	1.669	1.67	1.678	1.787
	Slope	0.188	0.191	0.197	0.202	0.207	0.214	0.218	0.218	0.221	0.224	0.228	0.23	0.231	0.333
	Intercept	-0.655	-0.72	-0.799	-0.897	-1.002	-1.082	-1.159	-1.205	-1.274	-1.304	-1.365	-1.395	-1.455	-2.581
MAR (70% women)	AAD	10.362	10.414	10.504	10.56	10.647	10.732	10.792	10.969	11.113	11.178	11.28	11.44	11.548	13.918
	SDAD	6.859	6.875	6.873	6.856	6.85	6.82	6.805	6.79	6.757	6.744	6.705	6.672	6.645	6.201
	BSD	1.378	1.375	1.41	1.404	1.423	1.407	1.443	1.458	1.473	1.493	1.5	1.513	1.511	2.298
	WSD	1.646	1.638	1.656	1.659	1.658	1.661	1.664	1.668	1.671	1.679	1.664	1.67	1.672	1.787
	Slope	0.178	0.181	0.18	0.185	0.186	0.187	0.192	0.198	0.2	0.202	0.204	0.208	0.211	0.333
	Intercept	-0.515	-0.539	-0.541	-0.591	-0.61	-0.663	-0.708	-0.799	-0.849	-0.887	-0.949	-1.005	-1.031	-2.581

Across different sample coverages (35–95%), the parameters’ values of age-mixing patterns in sexual partnership of infected individuals in MCAR scenario did not differ much with lower and higher sampling coverage. We can see in the Table 2 that the true values at 100% sampling coverage and those at different sampling coverage did not differ greatly except for the intercept and the slope. But, this is not the case for MAR scenario, notable differences were observed between the true parameters values at 100% sampling coverage and at lower coverage for all parameters.

## Discussion

In this simulation, we defined a priori higher age gap preference in sexual partnerships. From the results, we can see that in the transmission network constructed from the phylogenetic tree, age-mixing patterns in HIV transmission were depicted through proportions of phylogenetic pairings between men and women across different age groups; and the mean, and standard deviation of their age difference.

The true pairings Table 1, showed that, overall we had not many transmission clusters. This was due to the fact that we considered sampling at late stage of infection dynamic, 35–40 simulation time (2012–2017 calendar time) whereas the infection was introduced at 10 simulation time (1987 calendar time). In addition, starting 23 simulation time, there was ART interventions implemented which increased the chance to many people to start ART and become non-infectious. Furthermore, at 25, 28, 33, 36, 39 simulation times, eligibility to ART was improved by implementing early treatment through the increase of CD4 eligibility threshold, which increased the number of individuals on ART. Thus, having less infected individuals in that period of time (2012–2017) was supported by empirical evidence which proved that ART has the potential to decrease sexual transmission of HIV [39, 40]. The same table showed that, we had some age groups with zero median value of pairings, older women and younger men, this indicates that HIV transmission between these age groups was less likely to occur due to the configuration of age-mixing in sexual partnerships, and also due to ART interventions.

Women aged 25-39 years had the highest magnitude of the mean age difference (around 14 years on average). When we looked at their proportion of pairings, almost 68% of them were phylogenetically linked to men aged 40–49 years, and around 25% of these women were phylogenetically linked to men of the same age group (25–39 years). For men, the group of 40–49 years had the highest age difference magnitude (around 20 years), 70% (but the true value was around 50%) of their proportion of pairings were phylogenetically linked to younger women (15–24 years). The standard deviations of the age difference for younger women (15–24 years), women aged 25–39 years, and men aged 40–49 years were around 7, 5, and 3 years, respectively. Younger women (15–24 years) had a mean age difference around 13 years and more than 50% and 25% of them were phylogenetically linked respectively to men aged 25–39 years, and 40–49 years. More than 90% of men aged 25–39 years were phylogenetically linked to younger women (15–24 years). This showed that men aged 40–49 years were extensively connected to younger women (15–24 years), and with a non-negligible link to women between 25 and 34 years. Moreover, younger women (15–24 years) were also much connected to men aged 25–39 years who had a mean age difference around 11 years on average (between 10 and 12 years). The mean age difference for men aged 15–24 years was below 2 years and almost 100% of them were phylogenetically linked to younger women (15–24 years old). This showed that younger men could not play a significant role in cross-generational transmissions.

Given the mean and standard deviation of age difference across age groups, we can see that the higher age gap in sexual partnership presented in the Table 2 can be depicted in age difference of phylogenetic pairings between men and women in different age groups.

**Fig 7:**
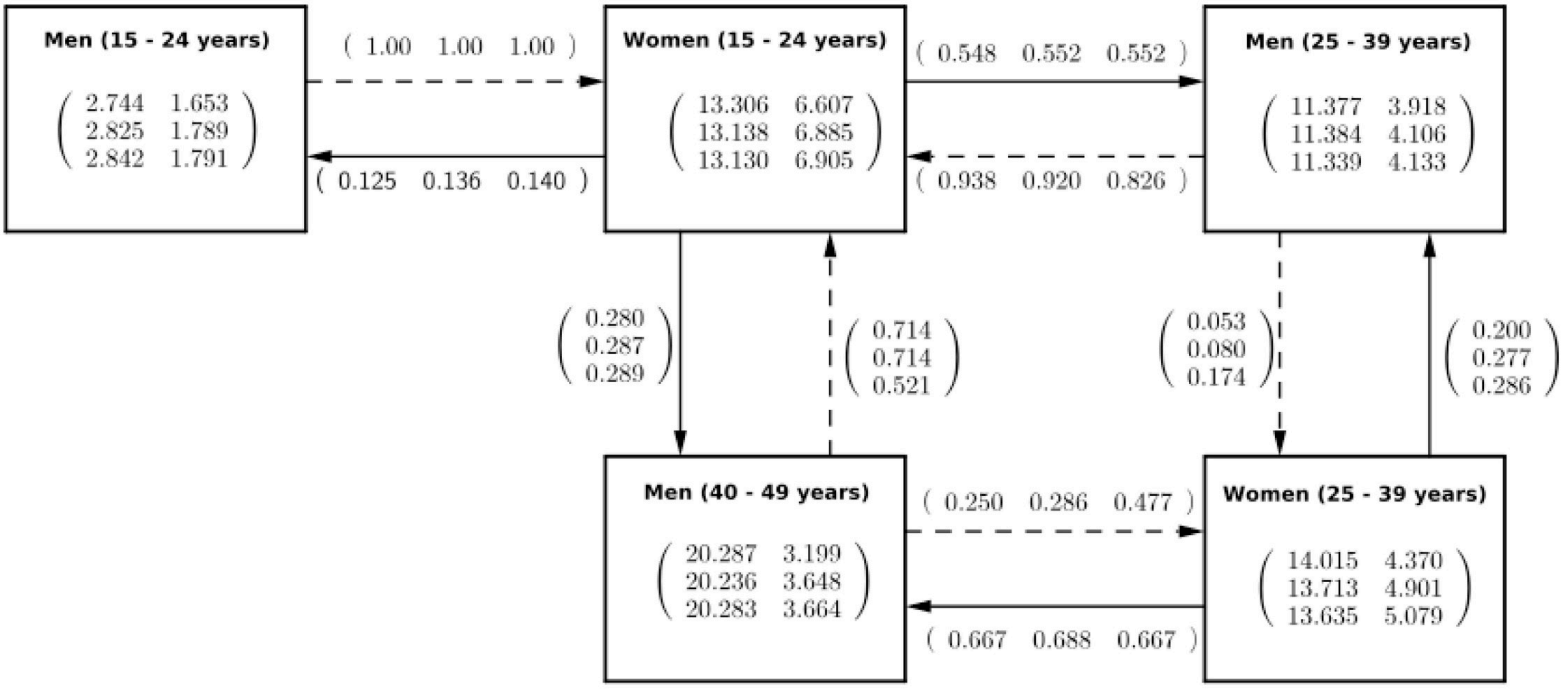
Representation of age-mixing patterns in HIV transmission. Solid and dashed arrows represent the proportions of women linked to men, and men linked to women respectively, the three values in each vector at these arrows are inferred proportions values at 55%, and 95% sampling coverage, and true value at 100%. The first column of matrices values represents the mean of age difference, and the second column represents standard deviation of age difference, between men and women who are phylogenetically linked.

Proportion values of phylogenetic pairings between men and women, together with mean and standard deviation of age difference, showed that younger women (15–24 years), women aged 25–39 years, older men aged 40–49 years, and men aged 25–39 years were key age groups to maintain a transmission cycle. But more importantly, younger women (15–24 years) and older men (40-49 years) were the main drivers of this transmission cycle. Referring to Fig 7, we can say that when younger men (15–24 years) grow-up and reach 25–39 years, higher proportion of them will be linked to younger women (15–24 years), same younger women to whom will be linked many older men (40–49 years). When younger women (15–24 years) grow-up and reach (25–39 years), many of them will be linked to older men (40–49 years).

The results proved that the proportions of phylogenetic pairings together with the means and standard deviations of the age difference between phylogenetically linked men and women could explain how younger women and women between 25 and 39 years together with older men (40–49 years) were the key age groups that would make the infection to persist. These findings showed that, when age-mixing patterns in HIV transmission exist in any population, we can be able to unveil these patterns through proportions of pairings between men and women, and age difference statistics across different age groups. These results are in agreement with evidence from empirical studies [13, 33], which proved that we can get insights on age-mixing patterns in HIV transmission by computing proportions of pairings; and mean, and standard deviation of age difference from phylogenetic trees.

Understanding the patterns of age-mixing in HIV transmission through a phylogenetic lens adds value to intervention design. Proportions of pairings show the magnitude of connectedness of pairs of HIV infections between different age groups. The mean, and standard deviation of the age gap between men and women (pairings) show on average the age difference between men and women in different age groups, and the magnitude of its dispersion. Concerns regarding age-mixing in HIV transmission come when the mean age difference is higher with narrow or wider standard deviation, and also when relatively small mean age differences occur with higher standard deviations.

The comparison of proportions of men phylogenetically linked to women showed that they were greatly below or above the true values even at higher sampling coverage. This implies that, we may be cautious in interpreting proportions of men phylogenetically linked to women because they maybe overestimated or underestimated when inferring them from transmission clusters computed from phylogenetic trees.

The comparison between estimates values obtained with MCAR and MAR scenarios at sampling coverages, showed that there were differences. Overall, the median values of proportions of pairings, and age difference statistics were from different distributions. Reported cases where we have same distributions, they were likely cases where we had sparse sampling.

When we compared the true parameter values of the age mixing patterns in sexual partnerships at 100% to those obtained at different sampling coverages (35%–95%) for MCAR, they were all relatively close. However, if we compared these true values at 100% to those obtained at different sampling coverages (35%–95%) for MAR, there were some notable discrepancies. This implies that, if we are able to depict age mixing patterns in HIV transmissions using conjointly proportions of phylogenetic pairings between men and women, and their age difference, it is worthy to consider the MCAR sampling strategy. Thus, for studies regarding age-mixing patterns in HIV transmissions with sequence data, we should avoid data collected from a part of the population where we may find a systematic imbalance of age and gender in the sample. We should not use sequence data from antenatal care (ANC) or sex-worker programs, but community survey data are encouraged. If we use sequence data from ANC or/and sex workers programs together with community data, we should take into account the age-gender imbalance in our results.

To estimate the proportions of pairings, on average a sampling coverage between 50% and 55% was sufficient in MCAR scenario, except for proportions of men where, even at 95% sampling coverage, the values were slightly below or above the true values. Thus, for proportions of women phylogenetically linked to men, and for the age gaps of men and women, we can use lower sample coverage, which makes such studies affordable in settings like SSA.

Our findings suggested that both sampling strategy, and sampling coverage have effect age-mixing estimates, and this support the finding from the study by Kusejko et al. [33] which proved that the mean age difference of pairs in the phylogenetic tree was influenced by sampling coverage. The comparison of the proportions and age gap estimates at different sampling coverages from MCAR and MAR (with at most 30%, 50%, and 70% women in the sample for any age group) with the Wilcoxon test showed that, in general there was a difference. Although we had sporadic scenarios where where we could not see differences, this might be due to gender imbalance, which made samples to become sparse. The same patterns in comparison was seen also for means and standard deviations of age differences. This may demonstrates one of the limitations of the study: for MAR, we assumed that we would obtain a certain percentage of women in the sample and across all age groups (e.g., 70% of women in all age groups: 15–24 years, 25–39 years, and 40–49 years), and it happened that we found small proportions of women or men in the populations due to the female:male ratio. This may be viewed as a technical artifact. We also have a limitation based on the population size: we could not increase the population size too much due to the limited computation time and memory available for the simulation, given the many simulations we intended to run.

## Conclusion

The proportions of phylogenetic pairings between men and women are important features of the age-mixing patterns in HIV transmission, as they show the magnitude of the connectedness between men and women in the transmission network across different age groups. The means and standard deviations of the age difference of men and women in these pairings across different age groups provide details on the magnitude of the age gap between infected individuals across all age groups in the transmission network. This is a kind of information that we can not obtain from any other source of data, except from phylogenetic trees. Transmission network being a subset of sexual network, hence, age difference in transmission clusters also reflected, to an extent, the sexual partnership age difference. For the sampling strategy, if sequences were missing completely at random, the results were more reliable. The more we increased the sequence coverage the more we improved the estimates; however, although the higher the sampling coverage the better estimates we get, we did not require higher coverage in order to have insight on age-mixing patterns in HIV transmission.

The main limitations of the study were mostly based on the fact that we used synthetic data with idealistic assumptions, particularly for molecular evolution. We know that certain evolutionary dynamics could change the structure of phylogenetic trees, which may change the inferences in reality. But, if we focus on between-host evolutionary dynamic, the results still informing us on age-mixing patterns in HIV transmission. The proportions of men and women phylogenetically linked together; and the means, and standard deviations of the age difference provided insights on the age mixing patterns in transmission. In real life, given the complexity of the transmission network based on social structures, these estimates may also tell a part of the story. These limitations together with artifacts due to the artificial age and gender imbalance in the MAR and the reduced population size can not be dismissed. Nevertheless, the approach we used to estimate HIV transmission network allowed us to investigate with improved information the age-mixing patterns in HIV transmission and cross-generational transmissions.

Therefore, the use of phylogenetic tree data has shown to be a good approach to assess age-mixing patterns in HIV transmission networks. By linking pairs of individuals in the transmission clusters, we can infer different age groups with close genetic relatedness. This approach informs us about the connectedness between men and women in the transmission network and the age difference between individuals.

## Supporting information

S1 Fig Point prevalence of HIV infection at 40 year simulation time. The point prevalence of HIV infection at 40 year of simulation time in an age- and gender-structured population.
(TIF)

S2 Fig Incidence of HIV infection in 35–40 years simulation time. The incidence of HIV infection in five years interval in an age- and gender-structured population.
(TIF)

S1 Appendix Simpact Cyan simulation model. Details about the simulation work-flow and parameters from events’ hazard functions and related settings.
(ZIP)
