# Supplementary figures and images for: Assessing the uncertainty around age-mixing patterns in HIV transmission inferred from phylogenetic trees
Source: PLoS One. 2021 Mar 25;16(3):e0249013. doi: 10.1371/journal.pone.0249013 (PMC7993798; doi:10.1371/journal.pone.0249013)

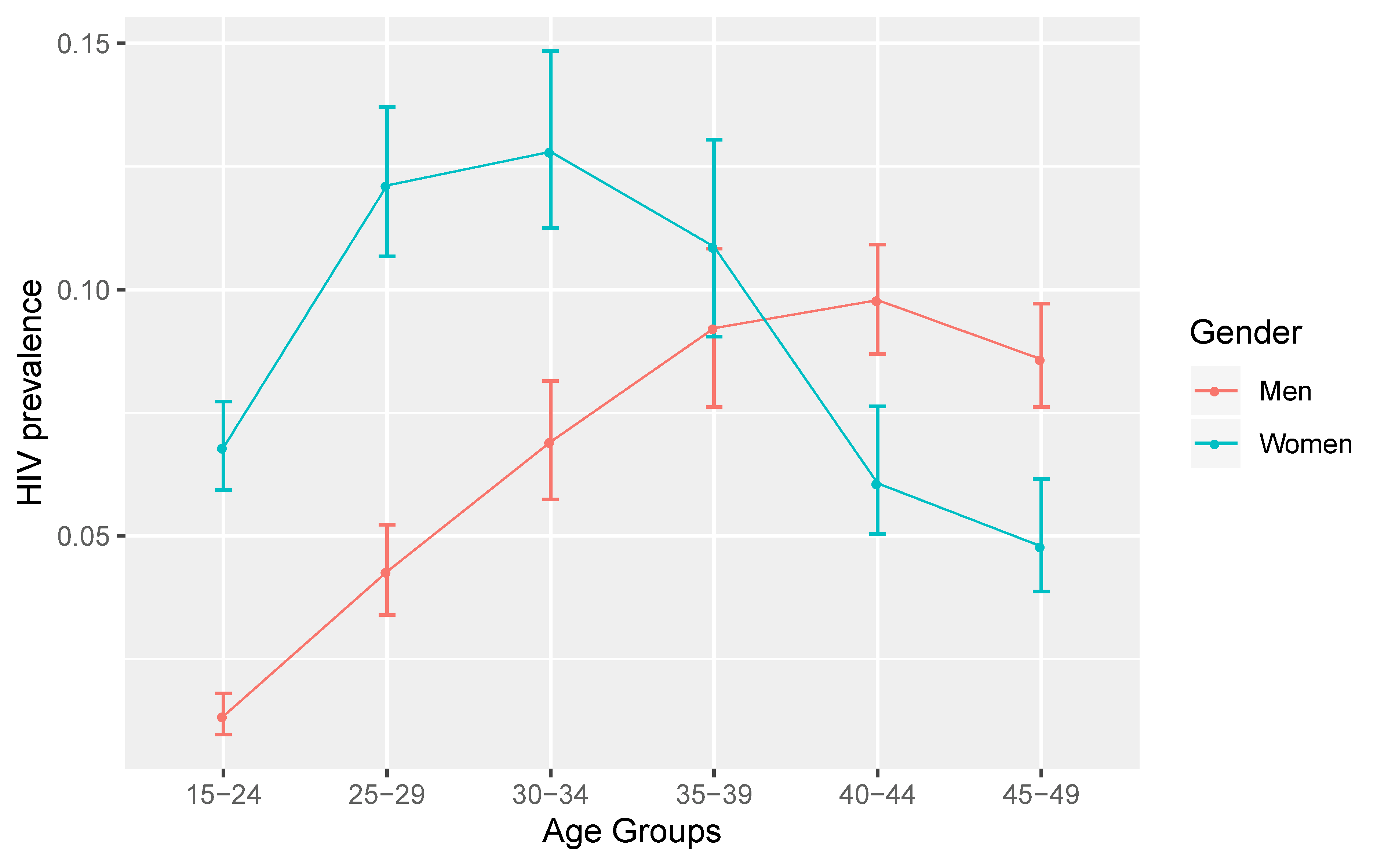

Supplement: S1 Fig — The point prevalence of HIV infection at 40 year of simulation time in an age- and gender-structured population. (TIF) [file pone.0249013.s001.tif]

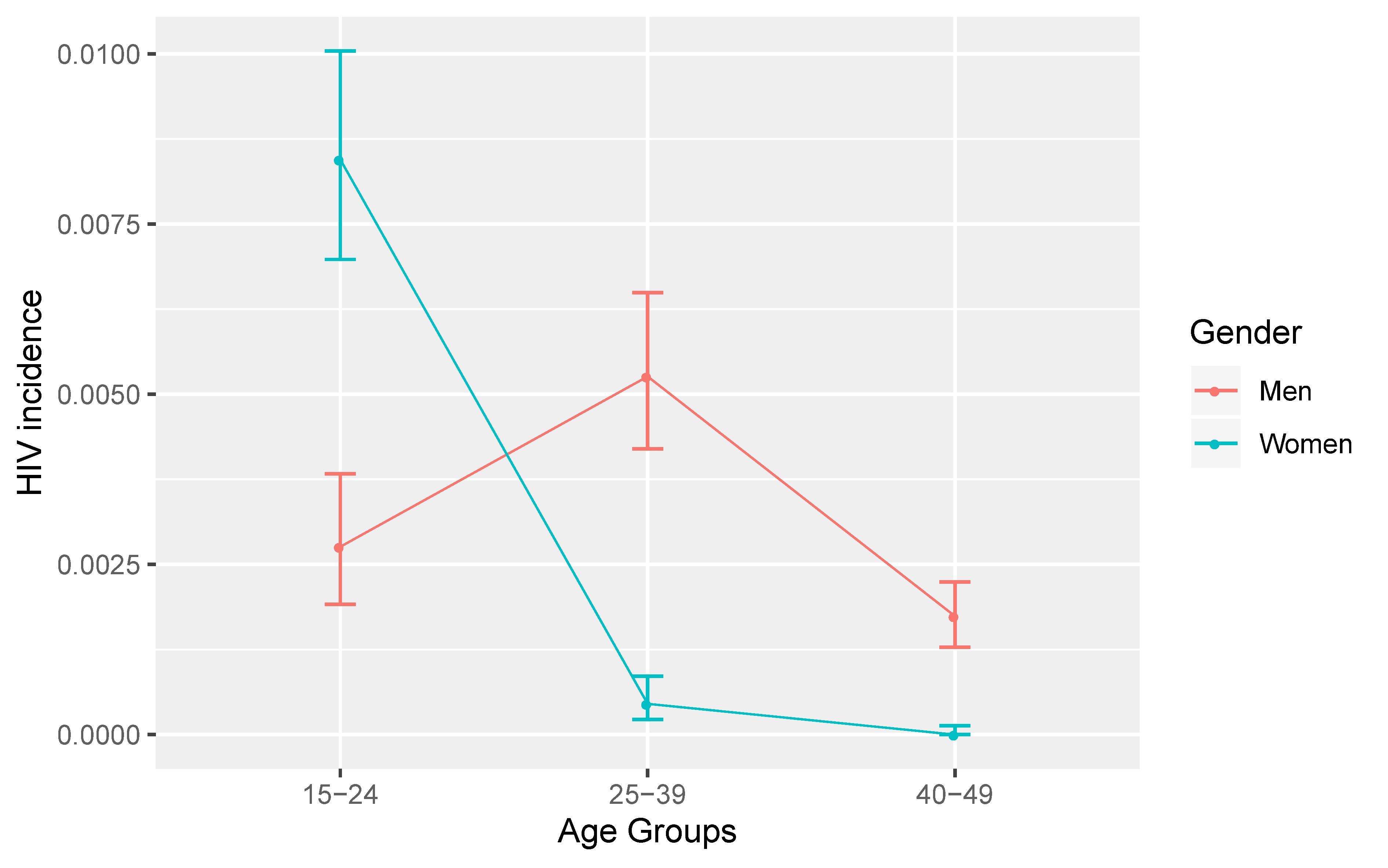

Supplement: S2 Fig — The incidence of HIV infection in five years interval in an age- and gender-structured population. (TIF) [file pone.0249013.s002.tif]
