# Supplementary material for: Assessing the uncertainty around age-mixing patterns in HIV transmission inferred from phylogenetic trees
Source: PLoS One. 2021 Mar 25;16(3):e0249013. doi: 10.1371/journal.pone.0249013 (PMC7993798; doi:10.1371/journal.pone.0249013)
Supplement: S1 Appendix — Details about the simulation work-flow and parameters from events’ hazard functions and related settings. (ZIP) [file pone.0249013.s003.zip › S1_Appendix.pdf]

Appendix: Simpact Cyan simulation model

1 Introduction

Before running Simpact Cyan, it might be configured based on simulation platform, and also the kind of sexual network to be simulated (heterosexual or same sex) across which HIV will be transmitted. To configure a simulation, you need to specify the options for which you want to use a value other than the default. Simpact Cyan has many parameters, as we can see in its documentation [1], and most of them were set given common knowledge, and findings from empirical studies. In this brief note, we provided description of parameters of interest which we tweaked for different events in the simulation model. More details are in the documentation of Simpact Cyan [1], where we extracted most of the content of this description note.

The simulation advances by figuring out which event should take place next, followed by executing code for that event. At the start, many initial events are typically scheduled, some set up to fire at a specific simulation time, some based on a hazard which may change during the simulation. During the simulation, new events will get scheduled, and some already scheduled events will be discarded (for example, in case someone dies, no other events involving this person will need to get executed anymore). The occurrence rates of events is calculated by evaluating the hazard functions for those events. Many of the parameters come from hazard functions, but there are also other parameters related to settings of events, more details are in the *hazard functions and parameters* section.

2 Configuration

The configuration of Simpact Cyan is mainly based on creating initial population, schedule initial events, and set parameters' values for events occurrence hazard functions, and related settings.

Create the initial population: give a number of men (*population.nummen*) and women (*population.numwomen*) who make the initial population. The population age is drawn from a defined Weibull survival distribution (shape = 5, scale = 65), in the absence of HIV-related

mortality, for the population at the start of the simulation. Depending on the debut age (15 years), people may be marked as being 'sexually active'.

Schedule the initial events: for each person, a 'normal' mortality event will be scheduled, and if needed, a debut event will be scheduled. Get the HIV epidemic started at some point, by scheduling an HIV seeding event. If specified, schedule the next simulation intervention (ART). This is a general way of changing simulation settings during the simulation. For pairs of sexually active persons, depending on the 'eyecap' settings ($population.eyecap.fraction$), schedule formation events. This parameter allows to specify with how many persons of the opposite sex (who are sexually active), specified as a fraction, someone can possibly have relationships.

Once the simulation is started, it will run either until the number of years specified in *population.simtime* have passed, or until the number of events specified in *population.maxevents* have been executed.

Hazard functions and parameters: Simpact Cyan uses mathematical expressions which allow the values of events' occurrence rates to depend on different factors which are known to influence the occurrence of events. For example, an event like relationship formation is affected by many factors such as age of the individual, ongoing sexual partnership, her/his age gap preference, among other, to capture their effect on the event's occurrence and to ensure that the hazard of occurrence is bounded between zero 0 and plus infinite ∞, the exponential function is a convenient candidate; hence, hazard functions are expressed as a linear expression raised to exponent.

$$h(t) = A \times exp^{B(t)} \tag{1}$$

Besides, events which occur based on their hazard functions, we have specific settings related to events. For example, distribution of age preference between partners, this is a relationship related setting, among others as we will see in subsequent sections.

3 Model parameters

The parameters, we choose which are in Table 1, were from the following hazard functions, and event related settings:

3.1 Sexual partnership event

$$
\begin{aligned}
\text{hazard}_{\text{rel}} &= F \times \exp\left(\alpha_{\text{baseline}}\right. \\
&+ \alpha_{\text{numrel,man}} P_{\text{man}} (1 + \alpha_{\text{numrel,scale,man}} g_{\text{man}}(t_{\text{ry}})) \\
&+ \alpha_{\text{numrel,woman}} P_{\text{woman}} (1 + \alpha_{\text{numrel,scale,woman}} g_{\text{woman}}(t_{\text{ry}})) \\
&+ \alpha_{\text{numrel,diff}} |P_{\text{man}} - P_{\text{woman}}| \\
&+ \alpha_{\text{meanage}} \left(\frac{A_{\text{man}}(t) + A_{\text{woman}}(t)}{2} \right) \\
&+ \alpha_{\text{dist}} |\vec{R}_{\text{man}} - \vec{R}_{\text{woman}}| \\
&+ \alpha_{\text{eagerness,sum}} (E_{\text{man}} + E_{\text{woman}}) + \alpha_{\text{eagerness,diff}} |E_{\text{man}} - E_{\text{woman}}| \\
&+ G_{\text{man}}(t_{\text{ry}}) + G_{\text{woman}}(t_{\text{ry}}) \\
&+ \left.\beta(t - t_{\text{ref}})\right)
\end{aligned}
$$

where

$$
\begin{aligned}
G_{\text{man}}(t_{\text{ry}}) &= [\alpha_{\text{gap,factor,man,const}} + \\
&\quad \alpha_{\text{gap,factor,man,exp}} \exp\left(\alpha_{\text{gap,factor,man,age}} \left(A_{\text{man}}(t_{\text{ry}}) - A_{\text{debut}}\right)\right)] \\
&\quad \times |g_{\text{man}}(t_{\text{ry}})|
\end{aligned}
$$

and

$$
\begin{aligned}
G_{\text{woman}}(t_{\text{ry}}) &= [\alpha_{\text{gap,factor,woman,const}} + \\
&\quad \alpha_{\text{gap,factor,woman,exp}} \exp\left(\alpha_{\text{gap,factor,woman,age}} \left(A_{\text{woman}}(t_{\text{ry}}) - A_{\text{debut}}\right)\right)] \\
&\quad \times |g_{\text{woman}}(t_{\text{ry}})|
\end{aligned}
$$

For sexual partnership event, the parameters we tweaked in the simulation were:

- $formation.hazard.agegapry.baseline$, the value of α_{baseline} in the expression for the hazard, allowing one to establish a baseline value.
- $formation.hazard.agegapry.gap.agescale.man$ controls $\alpha_{\text{gap,agescale,man}}$, which allows you to vary the preferred age gap with the age of the man in the relationship; and
- $formation.hazard.agegapry.gap.agescale.woman$ controls $\alpha_{\text{gap,agescale,woman}}$, which allows you to vary the preferred age gap with the age of the woman in the relationship.
- $formation.hazard.agegapry.numrel.man$, the value of $\alpha_{\text{numrel,man}}$ in the hazard formula, corresponding to a weight for the number of relationships the man in the relationship has; and

- $formation.hazard.agegapry.numrel.woman$, the value of $\alpha_{\text{numrel,woman}}$ in the hazard formula, corresponding to a weight for the number of relationships the woman in the relationship has.
- $formation.hazard.agegapry.numrel.diff$, the value of $\alpha_{\text{numrel,diff}}$ in the hazard expression, by which the influence of the difference in number of partners can be specified.
- $formation.hazard.agegapry.meanage$, the value of weight of $\alpha_{meanage}$ in the expression for the hazard, a weight for the average age of the partners.

3.2 Sexual partnership dissolution event

$$\begin{aligned}\text{hazard}_{\text{diss}} &= \exp\left(\alpha_0 + \alpha_1 P_{\text{man}} + \alpha_2 P_{\text{woman}} + \alpha_3 |P_{\text{woman}} - P_{\text{man}}|\right.\\ &\quad + \alpha_4 \left(\frac{(t - t_{\text{birth,man}}) + (t - t_{\text{birth,woman}})}{2}\right)\\ &\quad \left. + \alpha_5 |(t - t_{\text{birth,man}}) - (t - t_{\text{birth,woman}}) - D_{\text{pref}}| + \beta(t - t_{\text{ref}})\right)\end{aligned}$$

For sexual partnership dissolution event, the parameters we tweaked in the simulation were:

- *dissolution.alpha*.0, the value of α_0 in the expression for the hazard, allowing one to establish a baseline value.
- *dissolution.alpha*.1, the value of α_1 in the expression for the hazard, corresponding to a weight for the number of relationships the man in the relationship has.

3.3 Relationship related settings

Besides, defined factors mentioned at 3.1 which influence sexual partnership, it might depends also on individual eagerness, and age gap preference. Relationship settings define known facts that influence sexual partnership, and which are set by probability distributions in Simpact Cyan.

3.3.1 Eagerness

The eagerness of a person to form a relationship is used, to allow person's variation to engage in sexual partnership. Such an eagerness value can be defined for heterosexual relationships (*person.eagerness.man.dist.type* and *person.eagerness.woman.dist.type*)

and same sex relationships (*person.eagerness.man.msm.dist.type* and *person.eagerness.woman.wsw.dist.type*) independently, or a correlation

can be introduced by using a joint distribution ($person.eagerness.man.joint.dist2d$ and $person.eagerness.woman.joint.dist2d$). In the latter case, a pair of numbers is generated from a distribution, of which the first number is interpreted as the eagerness for a heterosexual relationship and the second for a same sex relationship. By default, independent random numbers are used, but this can be changed using the configuration value of $person.eagerness.man.type$ and $person.eagerness.woman.type$.

In our simulation the distribution type was a gamma distribution, thus, we tweaked the following parameters related to eagerness:

- $person.eagerness.man.dist.gamma.a$, represents α parameter for man's eagerness following a gamma distribution
- $person.eagerness.woman.dist.gamma.a$, represents α parameter for woman's eagerness following a gamma distribution
- $person.eagerness.man.dist.gamma.b$, represents β parameter for man's eagerness following a gamma distribution
- $person.eagerness.woman.dist.gamma.b$, represents β parameter for woman's eagerness following a gamma distribution

3.3.2 Age gap preference

Similarly to eagerness, preferred age gaps can be used in hazards, and these also can be defined for each person separately. Moreover, they can differ between heterosexual relationships: *person.agegap.man.dist.type* and *person.agegap.woman.dist.type*, and same sex: *person.agegap.man.msm.dist* and *person.agegap.woman.wsw.dist*.

In our simulation the distribution type was a normal distribution, thus, we tweaked the following parameters related to age gap preference:

- *person.agegap.man.dist.normal.mu*, for man's mean of normal distribution of age preference between men and women
- *person.agegap.woman.dist.normal.mu*, for woman's mean of normal distribution of age preference between women and men
- *person.agegap.man.dist.normal.sigma*, for man's standard deviation of normal distribution of age preference between men and women
- *person.agegap.woman.dist.normal.sigma*, for woman's standard deviation of normal distribution of age preference between women and men

3.4 HIV transmission event

When a relationship is formed between two people of which one is HIV infected, or when a relationship between two uninfected people exists and one of them gets infected, an HIV transmission event is scheduled. The hazard for this event is the following:

$$
\begin{aligned}
\text{hazard}_{\text{trans}} &= \exp\big(a + bV^{-c} + d_1 P_{\text{infected}} + d_2 P_{\text{uninfected}} \\
&+ \mathrm{W} f_1 \exp(f_2(A_{\text{woman}}(t_{\text{ry}}) - A_{\text{debut}})) \\
&+ e_1 HSV2_{\text{infected}} + e_2 HSV2_{\text{uninfected}} + g_1 b_{0\text{j}} + g_2 b_{1\text{j}}\big)
\end{aligned}
$$

For HIV transmission event, the parameters we tweaked in the simulation were:

- *hivtransmission.param.a* refers to the value of a in the expression for the hazard, providing a baseline value.
- *hivtransmission.param.b* refers to the value of b in the expression for the hazard. Together with the value of c this specifies the influence of the current viral load of the infected person.
- *hivtransmission.param.c* refers to the value of c in the expression for the hazard. Together with the value of b, this specifies the influence of the current viral load of the infected person.
- *hivtransmission.param.f*1 refers to the value of $f1$ in the expression of the hazard.
- *hivtransmission.param.f*2 refers to the value of $f2$ in the expression of the hazard. And by configuring the weights $f1$ and $f2$, is becomes possible to change the susceptibility of a woman depending on her age.

3.5 HIV infection monitoring event

When a person has been diagnosed as being infected with HIV, monitoring events are scheduled to follow up on the progress of the disease by inspecting the person's Cluster of Differentiation 4 (CD4) count. If this CD4 count is below the threshold set in *monitoring.cd4.threshold*, the person will be offered antiretroviral treatment. Depending on the person's willingness to accept treatment, treatment will then be started.

For HIV infection monitoring event, the parameters we tweaked in the simulation were:

- *monitoring.cd4.threshold* parameter for threshold set for the infected person to be offered antiretroviral treatment

- *monitoring.fraction.log.viralload* parameter to lower down the person's set-point viral load value if treatment is started

- *person.art.accept.threshold.dist.fixed.value* ART acceptance

3.6 Diagnosis event

When a person gets infected with HIV, either by transmission of the virus or by seeding the population to get the epidemic started, a diagnosis event will get scheduled. When fired, the person is deemed to feel bad enough to go to a doctor and get diagnosed as being HIV-infected. Upon diagnosis, a monitoring event will be scheduled very shortly afterwards, to monitor the progression of the disease and to offer treatment if eligible. This event is hazard-based, and the hazard is of the following form:

$$\begin{aligned}\text{hazard}_{\text{diag}} &= \exp(\text{baseline} + \text{agefactor} \times (t - t_{\text{birth}}) + \text{genderfactor} \times \text{G} \\ &\quad + \text{diagpartnersfactor} \times \text{P} + \text{isdiagnosedfactor} \times D + \beta(t - t_{\text{infected}}) \\ &\quad + \text{HSV2factor} \times \text{HSV2})\end{aligned}$$

For diagnosis event, the only parameter we tweaked in the simulation was:

- *diagnosis.baseline* controls the corresponding baseline value in the expression for the hazard

Other parameters were set to zero in our simulation, and details about them is in the documentation.

3.7 HIV infection stage and Viral load

When a person becomes HIV-infected, either by an HIV seeding event or because of transmission of the virus, a set-point viral load value is chosen and stored for this person. When a person receives treatment, the viral load is lowered (see the monitoring event) and if the person drops out of treatment the initially chosen set-point viral load is restored. The set-point viral load is the viral load that the person has during the chronic stage.

For further stages we have the following parameters, which were tweaked in our simulation:

- *person.vsp.toacute.x*: when the viral load during the acute stage is needed, it is determined in such a way that the transmission hazard increases by this factor, possibly clipped to a maximum value (*person.vsp.maxvalue*).

- $person.vsp.toaids.x$: when the viral load during the initial AIDS stage is needed, it is determined in such a way that the transmission hazard increases by this factor, possibly clipped to a maximum value ($person.vsp.maxvalue$).

- $person.vsp.tofinalaids.x$: when the viral load during the final AIDS stage is needed, it is determined in such a way that the transmission hazard increases by this factor, possibly clipped to a maximum value ($person.vsp.maxvalue$).

In any of the stage, when determining the viral load during acute, AIDS or final AIDS stages, a check is done so that the value does not exceed this maximum. If necessary, the calculated viral load value is clipped to this maximum value.

3.8 ART treatment dropout event

When a monitoring event gets triggered and the person is both eligible and willing to receive treatment, treatment is started causing the set-point viral load of the person to be lowered. When treatment starts, a dropout event is scheduled as well, to allow a person to drop out of treatment.

Currently, the dropout event is not hazard-based, instead a random number is picked from a one dimensional probability distribution as specified in *dropout.interval.dist.type* and related configuration options.

Using this configuration option you can specify the probability distribution to use when obtaining the time after which a person will drop out of treatment. By default, this is a uniform distribution with equal non-zero probability between 3 months and 10 years, and zero otherwise. Other distributions can be specified as well, as explained previously.

3.9 AIDS mortality event

When a person gets infected with HIV, an HIV-based time of death is determined. This time of death is determined as the time of infection plus the survival time as explained in the following formula [2]:

$$t_{\text{survival}} = \frac{C}{V_{\text{sp}}^{-k}} \times 10^{\text{x}}$$

where V_{sp} is the set-point viral load, x a parameter determined per person to allow randomness.

For AIDS mortality event, the parameters we tweaked in the simulation were:

- C is set in the model by $mortality.aids.survtime.C$

- k is set in the model by $mortality.aids.survtime.k$

By default, the survival time for a person after becoming HIV infected, is given by a simple relation based on the set-point viral load. Because an exact mapping from viral load to survival time is not that realistic, you can add some randomness to this relation using the distribution in $person.survtime.logoffset.dist.type$. When a person becomes infected, a random number is drawn from this distribution and will correspond to an offset in the survival time, as explained in the AIDS mortality event.

- $person.survtime.logoffset.dist.type$ one dimensional distribution can be used to add some randomness to the survival time until dying of AIDS related causes after becoming HIV infected.

3.10 Conception event

When a formation event has fired (so a man and a woman are in a sexual relationship), a conception event will be scheduled unless the woman is already pregnant. This is a hazard-based event, and its hazard at time t is defined as:

$$\begin{aligned}\text{hazard}_{\text{conc}} \quad = \quad & \exp(\alpha_{\text{base}} + \alpha_{\text{age,man}}\,(t - t_{\text{birth,man}}) + \alpha_{\text{age,woman}}\,(t - t_{\text{birth,woman}}) + \\ & \alpha_{\text{wsf}} \times \text{WSF} + (t - t_{\text{ref}})\beta)\end{aligned}$$

For conception event, the only parameter we tweaked in the simulation was:

- α_{base} set by *conception.alpha.base* baseline for conception hazard function

By default, only the α_{base} value is used ($conception.alpha_base$), resulting in a constant hazard, but other factors can be used as well: the age of the man and woman in the relationship can be taken into account using *conception.alpha.ageman* and *conception.alpha.agewoman*, the weekly sex frequency (WSF) using $conception.alpha.wsf$ and the 'age' of the relationship using conception.beta (tref is set to the time the relationship started). The value of WSF itself is currently chosen from the distribution specified in $conception.wsf.dist.type$, at the time the event gets scheduled.

Table 1. Values of selected parameters for the simulation of HIV epidemic.

Parameter	Explanation	Value
Initial configuration		
population.simtime	Simulation time	40
population.msm	Same sex sexual partnership	no
population.nummen	Initial men population	10 000
population.numwomen	Initial women population	10 000
hivseed.time	Time to introduce HIV in the population	10
hivseed.type	Consider amount not proportion of seed among population	
hivseed.amount	Amount of HIV seed individuals	10
hivseed.age.min	Minimum age for seed individual	20
hivseed.age.max	Maximum age for seed individual	50
debut.debutage	Age of being sexual active	15
population.maxevents	Maximum events to be simulated (beyond this number of events the simulation will stop, it can also stop with population.simtime)	1.2 *millions**
population.eyecap.fraction	Specify with how many persons of the opposite sex (who are sexually active), specified as a fraction, someone can possibly have relationships	0.2
Demographic		
birth.boygirlratio	boy/girl ratio	1.0/2.01
conception.alpha.base	Baseline for conception event	-3

Continued on next page ...

Table 1 – continued from previous page

Parameter	Explanation	Value
Sexual partnership		
person.eagerness.man.dist.gamma.a	α parameter for man eagerness following a gamma distribution	0.23
person.eagerness.woman.dist.gamma.a	α parameter for woman eagerness following a gamma distribution	0.23
person.eagerness.man.dist.gamma.b	*beta* parameter for man eagerness following a gamma distribution	45
person.eagerness.woman.dist.gamma.b	*beta* parameter for woman eagerness following a gamma distribution	45
person.agegap.man.dist.type	Distribution type for age gap preference for man	Normal
person.agegap.woman.dist.type	Distribution type for age gap preference for woman	Normal
formation.hazard.agegapry.baseline	Baseline for sexual partnership	2
person.agegap.man.dist.normal.mu	Mean for the normal distribution of age gap preference for man	10
person.agegap.woman.dist.normal.mu	Mean for the normal distribution of age gap preference for woman	10
person.agegap.man.dist.normal.sigma	Standard deviation for the normal distribution of age gap preference for man	5
person.agegap.woman.dist.normal.sigma	Standard deviation for the normal distribution of age gap preference for woman	5
$dissolution.alpha_0$	Baseline for relationship dissolution	−0.52
$dissolution.alpha_4$	Weight for the average age of the partners in relationship dissolution	−0.52

Continued on next page ...

Table 1 – continued from previous page

Parameter	Explanation	Value
HIV transmission		
hivtransmission.param.a	Baseline value for HIV transmission.	-1
hivtransmission.param.b	Influence of the current viral load of the infected person.	-90
hivtransmission.param.c	Influence of the current viral load of the infected person.	0.5
$hivtransmission.param.f1$	Weight for susceptibility of a woman depending on her age.	0.05
$hivtransmission.param.f2$	Weight for susceptibility of a woman depending on her age.	-0.14
HIV infection stages		
person.vsp.toacute.x	Viral load during the acute stage.	5
person.vsp.toaids.x	Viral load during the initial AIDS stage.	7
$person.vsp.tofinalaids.x$	Viral load during the final AIDS stage.	12
HIV infection monitoring		
$monitoring.fraction.log_viralload$	Lower the person's set-point viral load value when someone started ART.	0
ART interventions		
For ART intervention we have:		
time	Time for ART intervention	
diagnosis.baseline	Diagnosis baseline	
$monitoring.cd4.threshold$	CD4 count eligibility cutoff	
ART introduction 1:		
time		23 (2000**)

Continued on next page ...

Table 1 – continued from previous page

Parameter	Explanation	Value
diagnosis.baseline		-2
monitoring.cd4.threshold		100
ART introduction 2:		
time		25 (2002**)
diagnosis.baseline		-1.8
monitoring.cd4.threshold		150
ART introduction 3:		
time		28 (2005**)
diagnosis.baseline		-1.5
monitoring.cd4.threshold		200
ART introduction 4:		
time		33 (2010**)
diagnosis.baseline		-1
monitoring.cd4.threshold		350
ART introduction 5:		
time		36 (2013**)
diagnosis.baseline		-1
monitoring.cd4.threshold		500
ART introduction 6:		
time		39 (2016**)
diagnosis.baseline		-1
monitoring.cd4.threshold		700
person.art.accept.threshold.dist.fixed.value	ART acceptance	0.75

Continued on next page ...

Table 1 – continued from previous page

Parameter	Explanation	Value
dropout.interval.dist.type	Specification of ART dropout distribution	Uniform
dropout.interval.dist.uniform.min	Minimum value of the uniform dropout distribution	
dropout.interval.dist.uniform.max	Maximum value of the uniform dropout distribution	
AIDS mortality and survival		
mortality.aids.survtime.C	Relationship between set-point viral load and survival.	65
mortality.aids.survtime.k	Relationship between set-point viral load and survival.	-0.2
person.survtime.logoffset.dist.type	Type of distribution type for survival time randomness.	Normal
person.survtime.logoffset.dist.normal.mu	Mean of the normal distribution for survival time randomness.	
person.survtime.logoffset.dist.normal.sigma	Standard deviation of the normal distribution for survival time randomness.	
$**$ simulation time in calendar years, and $*$ 3 $\times$ simulation time $\times$ men initial population		

4 Assumptions for HIV transmission dynamics

Table 2. Main assumptions for the simulation of dynamic sexual partnership network and HIV transmission.

**Sub-component**	**Assumptions**
Demographic	Birth: when there is a sexual partnership formation, a conception event will be scheduled, after a conception event is triggered, a new birth event will be scheduled, so that the woman in the relationship will give birth to a new person a specific time, and the gender will be determined by the boy/girl ratio.<br>Mortality: normal mortality model follows Weibull distribution, and the time for AIDS mortality was determined as the time of infection plus the survival time.
Sexual partnership	We considered sexual partnerships such that the preferred age gap differed from one person to the next, but there was also an age dependent component in this preferred age gap, and we allowed for the weight of the age gap terms to be age dependent. We assumed the age gap to be normally distributed. The hazard function for partnership depended on the number of partners the man and woman in the relationship had. The debut age was set to be 15 years for men and women. Once a sexual partnership was established, it was subject to dissolution as well.

Continued on next page ...

Table 2 – continued from previous page

**Sub-component**	**Assumptions**
HIV transmission	Transmission is likely to occur when one individual in a sexual partnership is HIV infected. Transmission depends on the viral load level of an infected individual. A woman's susceptibility to HIV infection was considered to depend on her age.<br>Set-point viral load is the viral load that the person has during the chronic stage. In the acute stage or in the Acquired immunodeficiency syndrome (AIDS) stages, the configuration values $person.vsp.toacute.x$, $person.vsp.toaids.x$ and $person.vsp.tofinalaids.x$ cause the real viral load to differ from the set-point viral load in such a way that the transmission probability is altered: the hazard for transmission will increase by the factor x that is defined this way. We also assumed that once an infected individual is on ART, s/he cannot transmit the infection, thus, we set $monitoring.fraction.log_{v}iralload$ to 0.
ART interventions	If this CD4 count is below the threshold set in $monitoring.cd4.threshold$, the person will be offered antiretroviral treatment (ART). Depending on the person's willingness to accept treatment, treatment will then be started. We considered gradually increasing ART eligibility based on CD4 count thresholds as implemented in real life.
Disease progression	Follow up on the progress of the disease is done by inspecting the person's CD4 count. When a person receives treatment, the viral load is lowered and if the person drops out of treatment the viral load will increase.

5 HIV evolutionary dynamic

For HIV evolutionary dynamic, we considered population-level of variation of viral sequence of substitutions of nucleotide. We considered a forward-time approach, where the evolutionary history of infected population was simulated from the past to the present under $GTR + \Gamma + I$ substitution model. The root sequence was HIV-1 subtype C, it was retrieved from LOS ALAMOS database [3]. Model parameters (in Table 3) were estimated using jModelTest 2.1.3 [4], on large dataset of HIV-1 subtype C sequences from LOS ALAMOS.

Table 3. Parameters for HIV 1 substituion $GTR + \Gamma + I$ model

Name	Value
Relative Frequencies	
Adenine (A)	0.3857
Cytosine (C)	0.1609
Guanine (G)	0.2234
Thymine (T)	0.2300
Relative substitution rates	
r(A $\rightarrow$ G) = r(G $\rightarrow$ A)	2.9114
r(A $\rightarrow$ C) = r(C $\rightarrow$ A)	12.5112
r(A $\rightarrow$ T) = r(T $\rightarrow$ A)	1.2569
r(G $\rightarrow$ C) = r(C $\rightarrow$ G)	0.8559
r(G $\rightarrow$ T) = r(T $\rightarrow$ G)	12.9379
r(C $\rightarrow$ T) = r(T $\rightarrow$ C)	1.0000
Rate heterogeneity	
Shape parameter	0.9
Number of gamma rate categories	4
Fraction of invariant sites	
Proportion of invariant sites (I)	0.5230
Evolutionary rate	
Substitutions/site/year [1]	4.75×10^{-3}

We acknowledged that although consensus sequences of HIV remain useful for phylogenetic analysis at population level studies, there is high variability of the virus at within-host level in contrast with the low variability at population level [5]. In addition, many questions of evolutionary dynamics still remain unexplained up-to-date [6–8], hence, it still challenging to understand clearly HIV phylodynamics across scales [8–10]. However, the simplifications we made by only considering between-host variability, are in line with population-level transmission network analysis using standard phylogenetic methods [11].

References

1. Liesenborgs J. Simpact Cyan; 2017. https://simpactcyan.readthedocs.io/en/latest/index.html.

2. Arnaout RA, Lloyd AL, O'Brien TR, Goedert JJ, Leonard JM, Nowak MA. A simple relationship between viral load and survival time in HIV-1 infection. Proceedings of the National Academy of Sciences. 1999;96(20):11549–11553.

3. LANL. HIV-1 Subtype C; 2017. https://www.hiv.lanl.gov/components/sequence/HIV/asearch/query_one.comp?se_id=JN188292.

4. Darriba D, Posada D. jModelTest 2 Manual v0. 1.10. parallel computing. 2016;9(8):772.

5. Lemey P, Rambaut A, Pybus OG. HIV evolutionary dynamics within and among hosts. AIDS Rev. 2006;8(3):125–140.

6. Frost SD, Pybus OG, Gog JR, Viboud C, Bonhoeffer S, Bedford T. Eight challenges in phylodynamic inference. Epidemics. 2015;10:88–92.

7. Metcalf C, Birger R, Funk S, Kouyos R, Lloyd-Smith J, Jansen V. Five challenges in evolution and infectious diseases. Epidemics. 2015;10:40–44.

8. Gog JR, Pellis L, Wood JL, McLean AR, Arinaminpathy N, Lloyd-Smith JO. Seven challenges in modeling pathogen dynamics within-host and across scales. Epidemics. 2015;10:45–48.

9. Volz EM, Romero-Severson E, Leitner T. Phylodynamic inference across epidemic scales. Molecular biology and evolution. 2017;34(5):1276–1288.

10. Mideo N, Alizon S, Day T. Linking within-and between-host dynamics in the evolutionary epidemiology of infectious diseases. Trends in ecology & evolution. 2008;23(9):511–517.

11. Leigh Brown AJ, Lycett SJ, Weinert L, Hughes GJ, Fearnhill E, Dunn DT. Transmission network parameters estimated from HIV sequences for a nationwide epidemic. Journal of Infectious Diseases. 2011;204(9):1463–1469.
